# Beyond the genetics of flowering: Integration of ethylene signaling and histone methylation controls flowering time

**DOI:** 10.1093/plphys/kiad230

**Published:** 2023-04-17

**Authors:** Aida Maric

**Affiliations:** Plant Physiology, American Society of Plant Biologists, USA; CIBSS—Centre for Integrative Biological Signalling Studies, University of Freiburg, 79104, Freiburg, Germany; Plant Environmental Signalling and Development, Institute of Biology III, University of Freiburg, 79104 Freiburg, Germany

From the harsh winters in the arctic to the high mountains in the Himalayas, flowering plants have conquered the planet. Angiosperms adjusted to variable environments thanks to remarkable developmental plasticity. This developmental plasticity is marked by the ability of plants to constantly perceive and respond to signals from their environment. The constant environmental changes require a precise and fast response coordinated through a complex network integrating phytohormone signaling and epigenetic changes (Maury et al. 2019). Among other processes, timely response also regulates a hallmark event in plant life transition from vegetative to flowering phase. Although well studied, this phase of plant life represents a complex puzzle that combines endogenous and exogenous factors such as vernalization, circadian clock, day length, and phytohormone signaling (Campos-Rivero et al. 2017).

Numerous studies indicate the importance of hormonal crosstalk in the regulation of plant flowering (Campos-Rivero et al. 2017). Among phytohormones, ethylene is the only gaseous hormone produced in all plant tissues. These characteristics make it a versatile tool regulating different developmental processes such as fruit ripening, senescence, flowering time, as well as flower development (Iqbal et al. 2017; Feng et al. 2023). However, the reports of ethylene's role in flowering are conflicting because studies show ethylene signaling delays flower development in species such as Arabidopsis (*Arabidopsis thaliana*) and morning glory (*Pharbitis nil*), while treatment with exogenous ethylene promotes flowering transition in pineapple (*Ananas comosus*) and urn plant (*Aechmea fasciata*) (Iqbal et al. 2017).

In this issue of *Plant Physiology*, Xu et al. (2023) provide a link between ethylene signaling pathway and chromatin-based transcriptional control in the flowering Arabidopsis plant. The authors describe the role of ethylene signaling transcription factors ETHYLENE INSENSITIVE 3 (EIN3) and EIN3 LIKE 1 (EIL1) in direct recruitment of histone modification factor to regulate the expression of central floral repressor locus *FLOWERING LOCUS C* (*FLC*) and ultimately control ethylene-induced flowering delay in Arabidopsis.

Histone modifications include a range of changes at the histone tail, such as deposition of methyl or acetyl groups. Their ultimate function is to regulate gene expression by altering the chromatin state at specific loci. For example, when 2 methyl groups are deposited at the fourth lysine residue of histone 3 (H3K4me2), it results in chromatin relaxation and *FLC* gene activation (Figure). As previous studies reported, FLOWERING LOCUS D (FLD) removes methyl groups from the *FLC* locus, resulting in chromatin tightening, *FLC* gene repression, and promotion of flowering transition (He et al. 2003; Jiang et al. 2007; 2009) (Figure). Xu et al. (2023) report a delayed flowering phenotype for both *ein3 eil1* and *fld* mutants. Furthermore, using a combination of yeast 2-hybrid screening and in vivo co-immunoprecipitation assays, the authors show a direct interaction between EIN3/EIL1 and histone demethylase FLD. Because FLD regulates H3K4me2 levels at the *FLC* locus and EIN3/EIL1 complex directly interacts with FLD, Xu et al. (2023) moved on to examine the changes in H3K4me2 levels in plants misexpressing *ein3 eil1*. Indeed, the *ein3 eil1* double mutant as well as the *fld* mutant had higher levels of H3K4me2 at the *FLC* locus and a late flowering phenotype. Conversely, the methylation levels were downregulated in EIN3 and EIL1 overexpression lines. Taken together, these results are consistent with the idea that the *EIN3/EIL1* complex acts as a bridge to recruit FLD demethylase and regulate methylation state and the expression of *FLC* (Figure).

**Figure.**
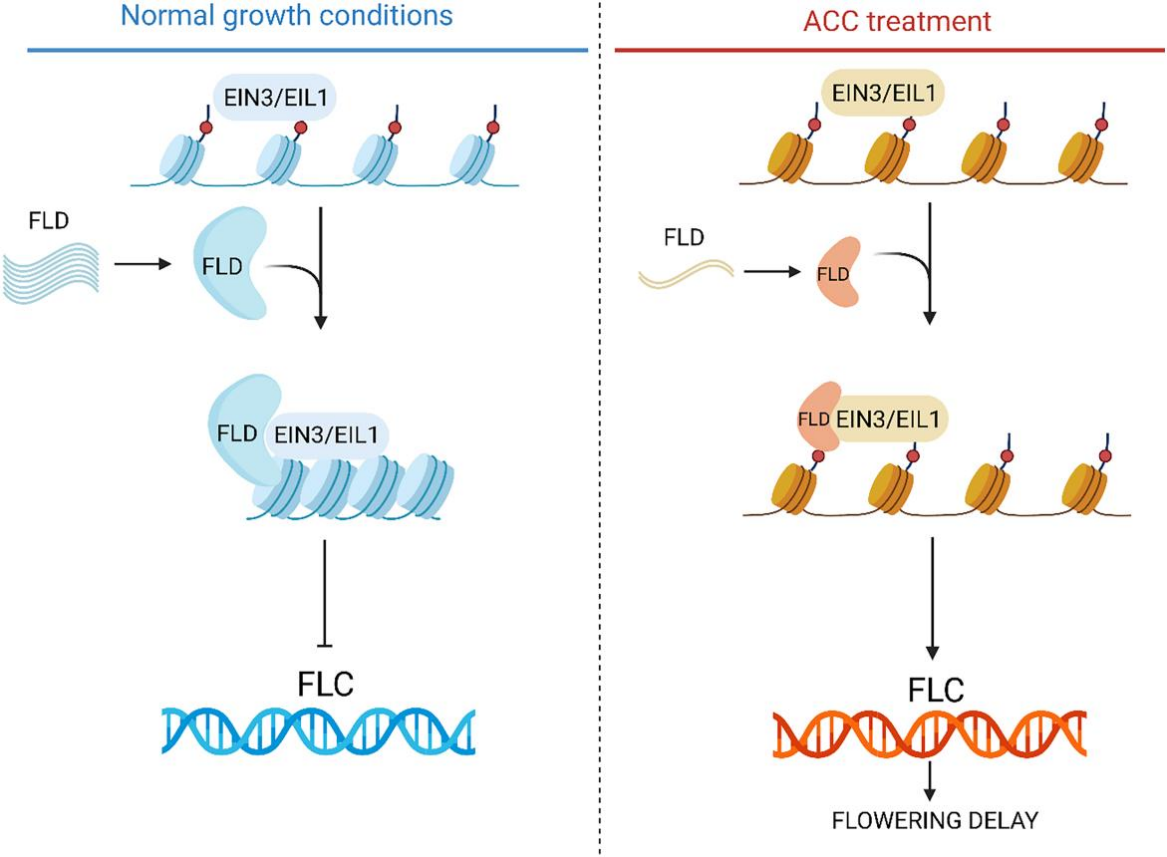
Schematic representation of ACC-dependent flowering repression in Arabidopsis. Ethylene signaling factors EIN3/EIL1 recruit FLD to control the expression of flowering repressor *FLC* and regulate transition to flowering time. Under ACC treatment, FLD availability for recruitment is reduced, leading to active transcription of *FLC*. (Figure modified from Xu et al. (2023) made by A.M. using BioRender.).

The ethylene signaling pathway is a well-studied network directly connecting environmental signals to gene expression regulation. Ethylene and its direct precursor, 1-aminocyclopropane-1-carboxylic acid (ACC), act as repressors of flowering (Patrick et al. 2007). Xu et al. (2023) provide a mechanism for this ACC-mediated flowering delay. Treatment of 10-day-old seedlings with ACC yielded 2 interesting results: on one hand, it increased the *FLC* expression levels, and on the other, it repressed the expression of *FLD*. Further chromatin immunoprecipitation (ChIP) experiments indicated that ACC-treated plants had higher H3Kme2 levels at the *FLC* locus due to lower levels of FLD availability. The H3Kme2 enrichment at the *FLC* locus causes enhanced *FLC* expression and flowering delay in ACC-treated plants (Figure). The ACC-mediated gene expression regulation proposed by Xu et al. (2023) represents an additional mechanism for plants to fine-tune their flowering response under constantly changing environmental conditions.

The role of phytohormones in plant growth and development cannot be overstated. Although individual hormones as well as their combinations have been studied within the context of plant development, there is still much to learn about their involvement in chromatin remodeling. The interaction between these 2 regulatory levels is not hierarchical and although we have plenty examples of phytohormones that regulate chromatin factors at the posttranslational level, it is plausible to say that biosynthesis of phytohormones is regulated by chromatin changes (Maury et al. 2019).

Having multiple pathways regulating flowering transition is a very important aspect of plant plasticity and fast response to environmental signals. Should a stressed plant delay reproduction until its hardships are over, or should it speed up its reproduction and try to produce seeds before it dies? Having multiple pathways regulating the transition to flowering allows for the implementation of different reproductive strategies under different environmental conditions. Ethylene, as a stress hormone, could serve as an integrator in the regulation of these different reproductive responses. Moreover, the discovery that the ethylene-associated regulation of flowering involves chromatin remodeling provides a new avenue of study for understanding the fine-tuned plasticity of the flowering regulatory network.
